# Pao Pereira Extract Attenuates Testosterone-Induced Benign Prostatic Hyperplasia in Rats by inhibiting 5α-Reductase

**DOI:** 10.1038/s41598-019-56145-z

**Published:** 2019-12-23

**Authors:** Jiakuan Liu, Tian Fang, Meiqian Li, Yuting Song, Junzun Li, Zesheng Xue, Jiaxuan Li, Dandan Bu, Wei Liu, Qinghe Zeng, Yidan Zhang, Shifeng Yun, Ruimin Huang, Jun Yan

**Affiliations:** 1 State Key Laboratory of Pharmaceutical Biotechnology and MOE Key Laboratory of Model Animals for Disease Study, Model Animal Research Center of Nanjing University, Nanjing, 210061 Jiangsu China; 2 Department of Comparative Medicine, Jinling Hospital, Nanjing University School of Medicine, Nanjing, 210002 Jiangsu China; 3 Shanghai Institute of Materia Medica, Chinese Academy of Sciences, Shanghai, 201203 China; 4 University of Chinese Academy of Sciences, Beijing, 100049 China; 5 Department of Bioscience and Bioengineering, School of Chemistry and Life Science, Jinling College of Nanjing University, Nanjing, 210061 Jiangsu China

**Keywords:** Prostate, Benign prostatic hyperplasia

## Abstract

Benign prostatic hyperplasia (BPH) is one of the most common diseases in the urinary system of elderly men. Pao extract is an herbal preparation of the bark of the Amazon rainforest tree Pao Pereira (*Geissospermum vellosii*), which was reported to inhibit prostate cancer cell proliferation. Herein we investigated the therapeutic potential of Pao extract against BPH development in a testosterone-induced BPH rat model. The administration of testosterone induced the prostate enlargement, compared with the sham operated group with vehicle treatment. The BPH/Pao group showed reduced prostate weight comparable with BPH/finasteride group. Notably, Pao treatment did not significantly reduce body weights and sperm number of rats, compared with the control group. Furthermore, Pao extract treatment reduced the proliferative index in prostate glands and testosterone-induced expression levels of AR, as well as androgen-associated proteins such as SRD5A1 and PSA. Moreover, Pao extract and its active component, flavopereirine, induced cytotoxicity on human prostate epithelial RWPE-1 cells in a dose- and time- dependent manner with G2/M arrest. Consistently, Pao extract and flavopereirine suppressed the expression levels of SRD5A1, AR and PSA, respectively. Together, these data demonstrated that Pao extract suppresses testosterone-induced BPH development through inhibiting AR activity and expression, and suggested that Pao extract may be a promising and relative safe agent for BPH.

## Introduction

Benign prostatic hyperplasia (BPH), an enlargement of the prostate gland, is a very frequent condition among older men. It is characterized by progressive overgrowth of both glandular and stromal tissues, causing an increase in prostate size^1,2^. The subsequent constriction of the urethra brings about lower urinary tract symptoms (LUTS), including urinary urgency, bladder outlet obstruction, and incomplete bladder emptying. By disrupting normal urination and disturbing consistent sleep, BPH reduces the quality of life of men who are afflicted^3^.

Age-related change in hormone balance of testosterone and dihydrotestosterone (DHT) is understood to be a major factor in the development of BPH^4–6^. Testosterone is converted into DHT by the action of 5α-reductases, the enzymes involved in steroid metabolism. Since DHT possesses 3-fold higher affinity for androgen receptor (AR) than testosterone, it is an important mediator of BPH development^5,6^. When men grow older, the enzymatic activity of 5α-reductases and transactivation activity of AR are prone to increase because of the imbalance of androgen^7^. Activation of AR through binding of DHT promotes prostate cell proliferation and survival^8–12^. In addition, high DHT level also raises the levels of prostate specific antigen (PSA) by inducing the transactivation activity of AR^7,8^.

Inhibitors of 5α-reductases (5αRIs, *e.g*. finasteride) are commonly used for the BPH treatment because they reduce the level of DHT^13^. Another BPH therapeutic agent, α1-adrenoceptor antagonists (such as α-blockers) primarily target the smooth muscles in the bladder neck and prostate, resulting in the relaxation of the smooth muscles and the subsequent alleviation of symptoms^14^. Unfortunately, the intakes of both 5αRIs and α-blockers will lead to several side effects, such as erectile dysfunction and cardiovascular risks, which limit their uses in clinical practice^15–17^. On the contrary, herbal medicines are regarded as a useful alternative approach for BPH treatment because patients often discern that certain plant extracts can have similar efficacy with milder side effects. In several European countries, phytomedicines comprise approximately 50% of all BPH treatment, whereas 40% of American men choose to use herbal medicine to treat BPH^18,19^. Indeed, a couple of extracts from plants, including *Serenoa repens* (saw palmetto)*, Pygeum africanum* (pygeum) and *Secale cereal* (rye), have been tested in current clinical trials for treating BPH^20–22^.

Pao pereira extract is an herbal extract derived from the bark of the Amazon rainforest tree, *Geissospermum vellosii*, that has been used historically as a medicine by South American Indian tribes. The bark of Pao pereira shows anti-plasmodial, antinociceptive and anti-inflammatory activities^23,24^. Recent studies revealed that a β-carboline alkaloid-enriched Pao extract has inhibitory effects on two prostate cancer lines, LNCaP and PC3 by inhibiting cell proliferation and survival^25,26^. Moreover, Pao extract can also chemosensitize ovarian cancer cells to carboplatin and pancreatic cancer cells to gemcitabine, respectively^27–29^. Given that BPH is caused by increased proliferative rates of prostate epithelial and stromal cells, we hypothesize that Pao extract can suppress BPH development by inhibiting prostate cell proliferation. Herein, we investigate the activities of Pao pereira extract against BPH and dissect its underlying mechanisms. One of its active components, flavopereirine, is also tested.

## Results

### Pao extract prevents testosterone propionate (TP) induced BPH in a rat model

**Figure 1:**
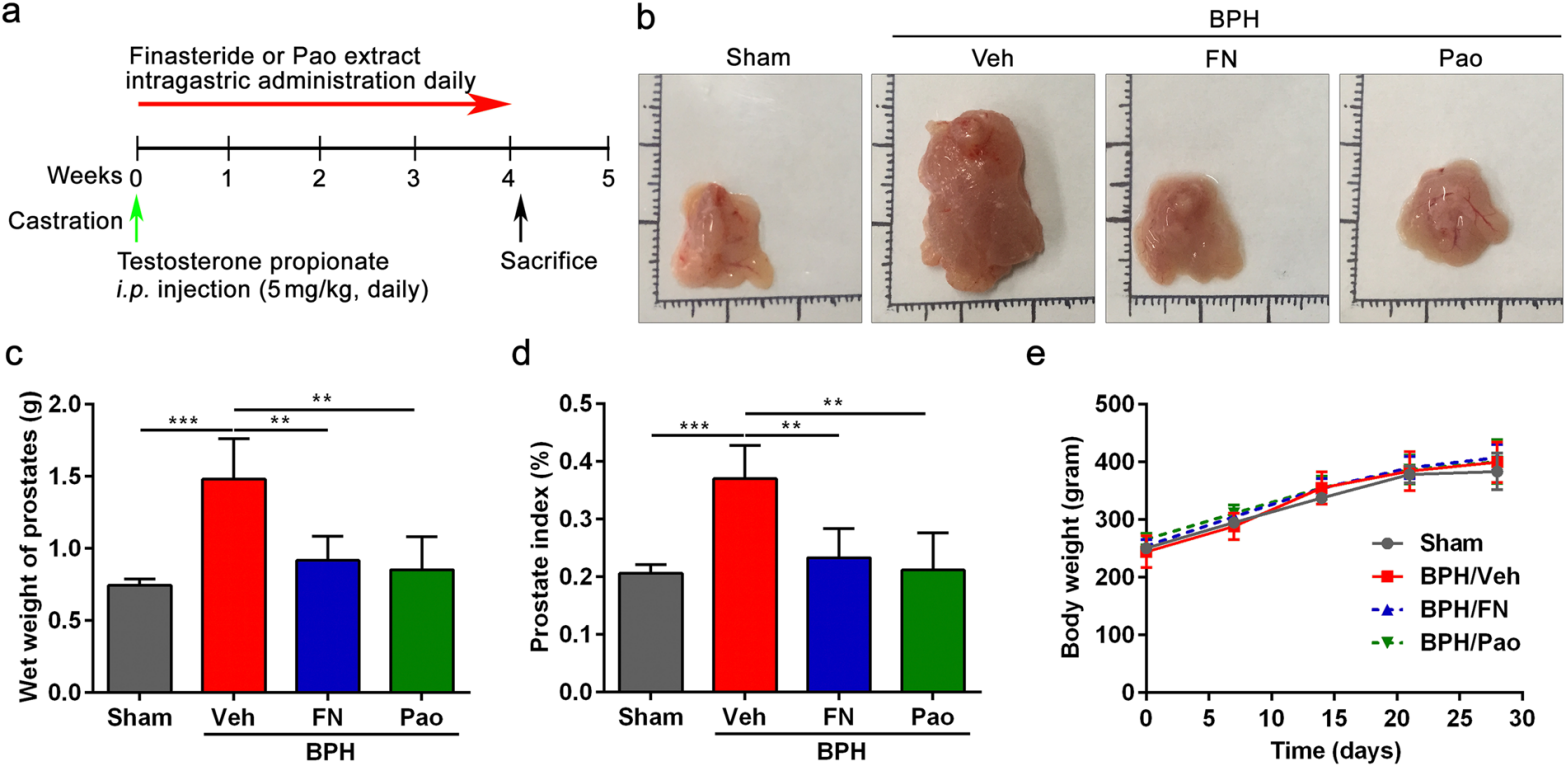
Effects of Pao extract on rat prostate. (**a**) Schematic presentation of experimental procedure. Sham group as control group: After sham operation, rats were treated with *i.p*. injection of corn oil and oral saline; BPH/Veh group: After castration, rats were *i.p*. injection of 5 mg/kg testosterone propionate (TP) daily and were intragastric administrated with saline; BPH/FN (Finasteride) and BPH/Pao groups: After castration, rats were *i.p*. injection of 5 mg/kg TP and intragastic administration of finasteride (10 mg/kg) or Pao extract (20 mg/kg) daily for 28 days, respectively. (**b**) The representative photos of the dissected prostate glands from four groups. (**c**) The weight of whole prostate without urethra. (**d**) The changes in the rat prostate index of four groups. (**e**) Effect of Pao extract on body weight. n = 5; **p < 0.01; ***p < 0.001.

The BPH rat model was established as shown in Fig. 1a. The castrated rats were treated with testosterone propionate (TP), and the finasteride treatment was used as a positive control and sham group is referred to as control. After 28 days, the prostates were collected and weighed (Fig. 1b,c). As expected, the wet weight and mean prostate index in the BPH group (BPH/Veh group) was significantly elevated compared with that in the control group (Sham group). Interestingly, administration of either Pao extract (BPH/Pao group) or finasteride (BPH/FN group) significantly reduced the wet weight of prostate and prostate index in BPH rats (Fig. 1c,d). In addition, there were no significant differences in the body weight of rats among groups (Fig. 1e). These results suggest the anti-BPH efficacy of Pao extract *in vivo*.

### Pao extract does not reduce sperm count in rats

Since reduced sperm count is one of the serious side effects of finasteride in rats and human^30,31^, we wanted to examine whether Pao extract has milder side effects compared with finasteride. Rats were treated with vehicle, finasteride and Pao extract for 28 days, respectively. After treatment, the sperm counts were analyzed in vehicle, finasteride and Pao extract treatment groups. Consistent with other reports, finasteride significantly reduced the sperm count compared to vehicle treatment^32^, while there were no significant differences between Pao and vehicle treatment groups (Fig. S1a,b). These results indicate that Pao extract has milder side effects compared to finasteride.

### Pao extract inhibits the multiplication of prostate epithelial cells in BPH rat model

**Figure 2:**
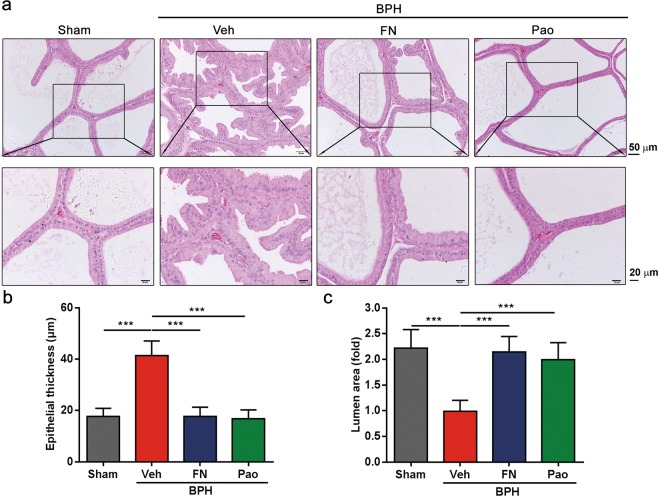
The histopathological analysis of the prostate tissues in the testosterone-induced BPH rats after being treated with Pao extract. (**a**) H&E staining of the sham control, BPH/Veh, BPH/FN and BPH/Pao groups. The sections were photographed by microscope. The scale bars in photos above are 50 μm and in photos below are 20 μm. (**b**) Quantification of the thickness of epithelial layers; (**c**) quantification of the fold changes of the lumen areas among four groups. ***p < 0.001.

It has been reported that TP treatment can induce prostatic hyperplasia in rats, which was manifested as a significant thickening of the prostatic epithelial cell layer and reduction of lumen area in the acini^33,34^. The histological staining showed that the thickness of the prostatic epithelial cell layer in the BPH/Veh group significantly increased compared with that of the sham control group. On the contrary, Pao extract treatment significantly reduced the thickness (Fig. 2a,b). Consistently, in comparison with the sham control group, the lumen areas of BPH group were decreased, which was reversed by Pao extract treatment and finasteride treatment, respectively (Fig. 2c). This suggests that Pao extract can restrict the growth of prostate cells in a testosterone-induced BPH rat model.

**Figure 3:**
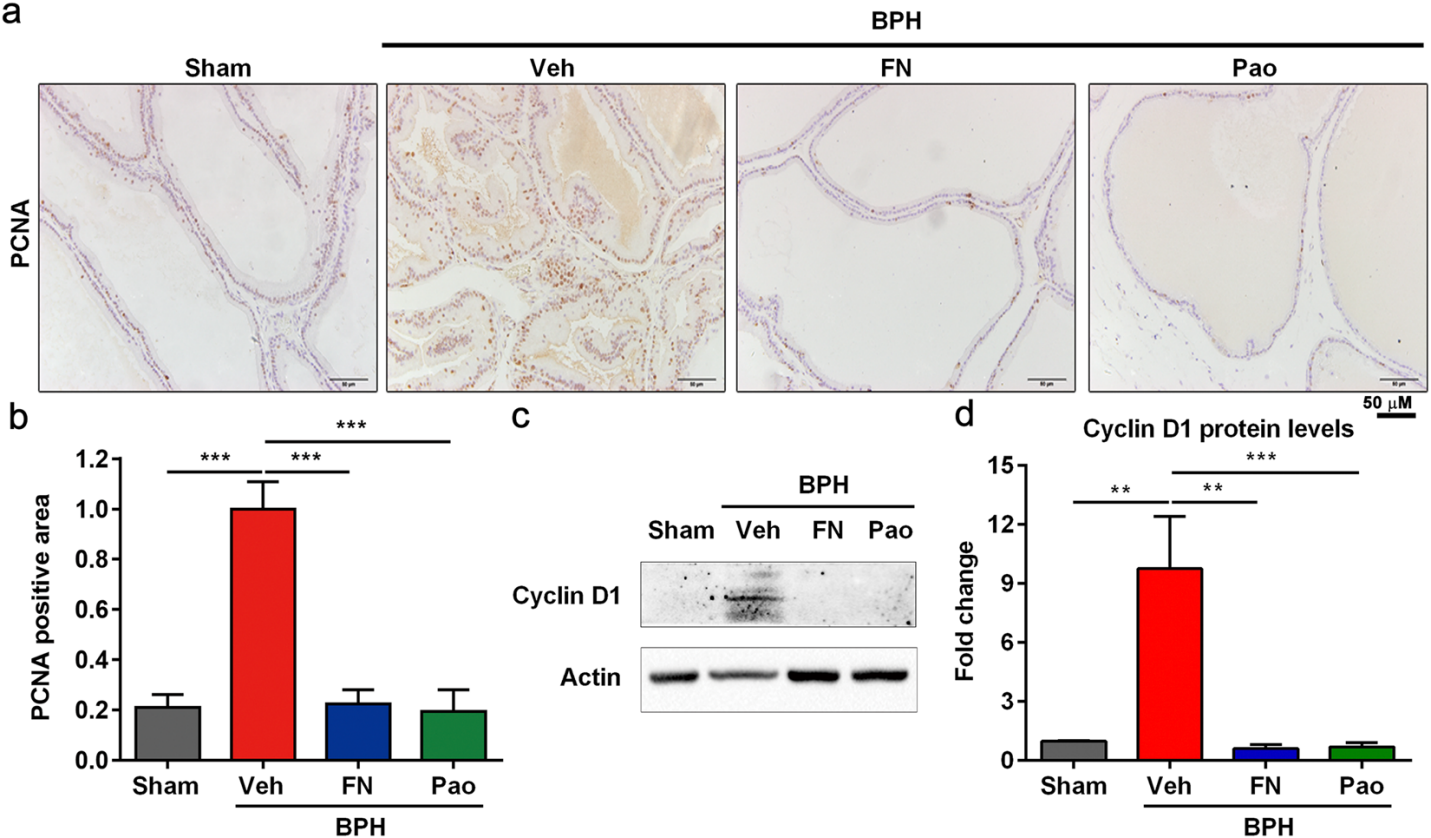
Pao extract treatment inhibited cell proliferation in prostate. (**a**) IHC staining of PCNA on Sham control, BPH/Veh, BPH/FN and BPH/Pao groups. Scale bar, 50 μm. (**b**) Quantification of PCNA positive areas of IHC staining from four groups. (**c**) Western blot of Cyclin D1 protein level in rat prostate tissues. (**d**) Quantification of Cyclin D1 protein level in rat prostate tissues. The values were presented as the mean ± SD of three independent experiments. **p < 0.01; ***p < 0.001.

To further confirm the reduction of prostate cell proliferation by Pao extract treatment, we carried out IHC staining to examine the level of a proliferative biomarker–proliferating cell nuclear antigen (PCNA). The percentage of PCNA-positive cells in the prostate glands significantly and dramatically increased in BPH/veh group compared with sham control group, but such change was significantly reversed by Pao extract (Fig. 3a,b). Consistently, the levels of Cyclin D1, another proliferative marker, underwent a similar reduction in response to Pao extract (Fig. 3c,d). Overall, these results indicate that Pao extract can effectively inhibit the multiplication of prostate epithelial cells in the rats with BPH induced by TP.

### Pao extract inhibits AR expression

**Figure 4:**
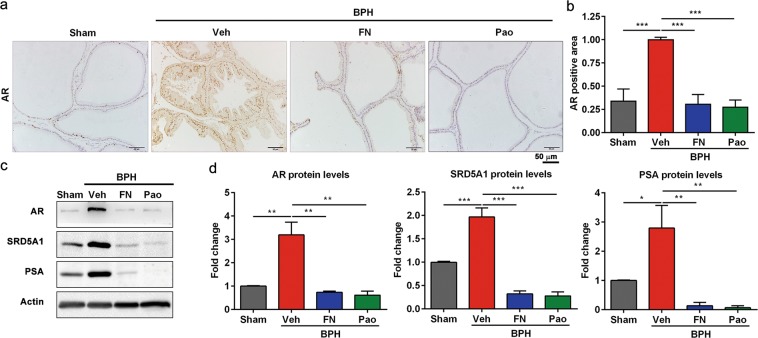
Pao extracts downregulated the expression of AR, SRD5A1, and PSA. (**a**) IHC staining of AR in the sham control, BPH/Veh, BPH/FN and BPH/Pao groups. Scale bar, 50 μm. (**b**) Quantification of AR positive area in four groups. (**c**) Western blot analysis of AR, SRD5A1, and PSA protein expression in rat prostate tissues. (**d**) Quantification of AR, SRD5A1, and PSA protein expression levels in rat prostate tissues from four groups by Image J software. The values were presented as the mean ± SD of three independent experiments. *p < 0.05; **p < 0.01; ***p < 0.001.

Androgen receptor (AR) has been implicated in BPH development and plays an important role in prostate cell proliferation and survival^5,6^. To investigate whether Pao extract treatment can suppress AR-associated pathway, we detected the AR level in four experimental groups. As shown in Fig. 4a,b, the nuclear AR levels in prostate epithelial cells were elevated in BPH/veh group, whereas both finasteride and Pao extract treatment significantly reduced AR levels, respectively. Furthermore, the levels of 5α reductase (SRD5A1), AR and AR downstream target PSA were induced by testosterone treatment in BPH/veh group. However, these increases were significantly reduced by Pao extract treatment and finasteride treatment, respectively (Fig. 4c,d). Taken together, Pao extract can suppress AR-associated pathway in the prostate epithelial cells in BPH model.

### Pao extract inhibits RWPE-1 human prostate epithelial cell proliferation

**Figure 5:**
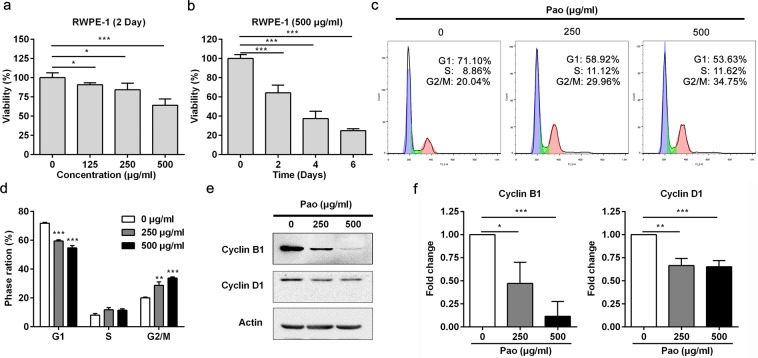
Pao extract inhibits cell proliferation and induces cell cycle arrest in human prostate RWPE-1 cells. (**a**) RWPE-1 cells were treated with Pao extract with the indicated concentrations for 48 h and the cytotoxicity was evaluated by the MTT assay. (**b**) RWPE-1 cells were treated with 500 μg/ml Pao extract for 0, 2, 4, and 6 days and evaluated by the MTT assay. (**c**) Flow cytometry analysis of RWPE-1 cells treated with vehicle and Pao extract with the indicated concentrations. (**d**) Quantification of percentage of each cell cycle in vehicle, 250 and 500 μg/ml of Pao extract. (**e**) Western blotting assay of RWPE-1 cells treated with Pao extract. Actin was used as a loading control. (**f**) The semi-quantitative analysis of Western blotting data in (**e**) by densitometry using Image J software. The values were presented as the mean ± SD of three independent experiments. *p < 0.05; **p < 0.01; ***p < 0.001.

The elevated proliferation rates of prostate epithelial cells are frequently detected in human BPH samples, as well as in BPH rat model. To determine whether Pao extract can efficiently inhibit human prostate epithelial cells, we utilized one human prostate epithelial cell line, RWPE-1. As shown in Fig. 5a, Pao extract treatment inhibited RWPE-1 cell proliferation in a dose-dependent manner. 500 μg/ml Pao extract signficantly suppressed cell viablity by almost 30% after the 48 h-treatment. Moreover, we also detected that 500 μg/ml Pao extract significantly and strikingly inhibited RWPE-1 cell proliferation in a time-dependent manner, with almost 70% inhibitory effect after 6 days (Fig. 5b).

### Pao extract induces cell cycle arrest in RWPE-1 cells

To further dissect how Pao extract inhibits cell proliferation, we treated RWPE-1 cells with Pao extract for 48 h, followed by the cell cycle analysis. As shown in Fig. 5c, we found that Pao extract induced G2/M cell cycle arrest in a dose-dependent manner. Pao extract significantly increased the percentage of G2/M cell population from 20.11 ± 0.26% of vehicle control group to 28.75 ± 1.41% of 250 μg/ml and 33.74 ± 0.53% of 500 μg/ml Pao extract group, respectively (Fig. 5d). Biochemical analysis confirmed that Cyclin B1 and Cyclin D1 expression levels were significantly reduced in RWPE-1 cells treated with Pao (Fig. 5e,f).

### Pao extract inhibits AR, PSA and SRD5A1 expression in RWPE-1 cells

**Figure 6:**
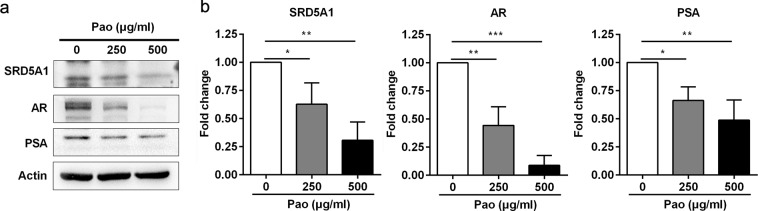
Pao extract inhibits AR, PSA and SRD5A1 expression in RWPE-1 cells. (**a**) Western blotting assay of RWPE-1 cells treated with Pao extract with the indicated concentrations. Actin was used as a loading control. (**b**) The semi-quantitative analysis of Western blotting data in (**a**) by densitometry using Image J software. The values were presented as the mean ± SD of three independent experiments. *p < 0.05; **p < 0.01; ***p < 0.001.

To further corroborate whether Pao extract can inhibit the expression of SRD5A1, AR, and PSA levels in RWPE-1 cells, we treated RWPE-1 cells with Pao extract. Consistent with the results in BPH rat model, Pao extract significantly suppressed AR, PSA and SRD5A1 in RWPE-1 cells in a dose dependent manner (Fig. 6a,b). Overall, our data demonstrated for the first time that Pao extract can efficiently inhibit BPH pathogenesis, at least partially through suppressing SRD5A1, AR and PSA expressions in prostate epithelial cells.

### Flavopereirine perchlorate (Fla) inhibits the proliferation of RWPE-1 cells

**Figure 7:**
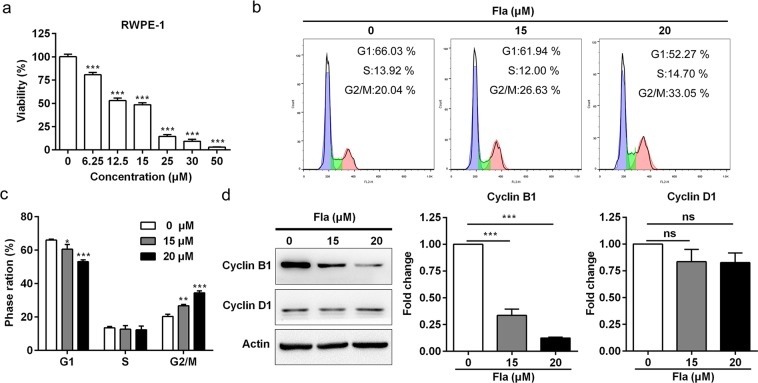
Flavopereirine inhibits cell proliferation and induces cell cycle arrest in RWPE-1 cells. (**a**) RWPE-1 cells were treated with flavopereirine with different concentrations for 48 h by the MTT assay. (**b**) Cell cycle analysis of RWPE-1 cells treated with vehicle and Fla with the indicated concentrations. (**c**) Quantification of percentage of each cell cycle in vehicle, 15 and 20 μM of Fla. (**d**) Western blotting assay of RWPE-1 cells treated with Fla. Actin was used as a loading control. (**e**) The semi-quantitative analysis of Western blotting data by densitometry using Image J software. The values were presented as the mean ± SD of three independent experiments. Fla, Flavopereirine. *p < 0.05; **p < 0.01; ***p < 0.001.

Pao extract is a β-carboline alkaloids-enriched extract and the active components of the same genus of Pao were β-carboline alkaloids and indole alkaloids^27^. Flavopereirine (Fla) alkaloid isolated from Pao is a pharmaceutical important alkaloid, which possesses several biological activities, such as DNA damaging, antiplasmodial activity and cytotoxicity^35–37^. In order to dissect whether Pao extract inhibits the BPH through Fla, we used Fla to treat RWPE-1 cells. As shown in Fig. 7a, Fla inhibited the proliferation of RWPE-1 cells in a dose-dependent manner. We also analyzed cell cycle of RWPE-1 cells treated with Fla by flow cytometry. The results showed that Fla induced G2/M cell cycle arrest in a dose-dependent manner (Fig. 7b,c). The expression levels of Cyclin B1 was significantly reduced in RWPE-1 cells treated with Fla without any change of the level of Cyclin D1 (Fig. 7d).

### Fla inhibits the expression of AR, PSA and SRD5A1 in RWPE-1 cells

**Figure 8:**
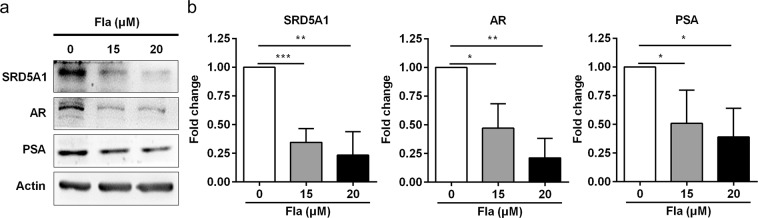
Flavopereirine inhibits AR, PSA and SRD5A1 expression in RWPE-1 cells. (**a**) Western blotting assay of RWPE-1 cells treated with Fla. (**b**) The semi-quantitative analysis of Western blotting data in (**a**) by densitometry using Image J software. The values were presented as the mean ± SD of three independent experiments. *p < 0.05; **p < 0.01; ***p < 0.001.

To further confirm whether Fla could inhibit the expression of SRD5A1, AR, and PSA in RWPE-1 cells, we treated RWPE-1 cells with Fla. Fla could also significantly suppressed protein level of AR, PSA and SRD5A1 in RWPE-1 cells in a dose dependent manner (Fig. 8a,b). Taken together, these results demonstrated that Pao extract could inhibit the proliferation and the expression of SRD5A1, AR and PSA of RWPE-1 cells, at least partially through Fla.

## Discussion

Pao extract, derived from bark of Amazonian tree Pao Pereira, is commonly used in South American medicine to treat a variety of ailments including cancer^23,24^. We and others have reported that Pao extract can inhibit human prostate cancer cell proliferation and survival *in vitro* and *in vivo*^25,26^. However, there is no evidence on its effects on BPH. Herein we examined the therapeutic effects of Pao extract on BPH for the first time and found that Pao extract and one of its component, flavopereirine (Fla) can significantly inhibit SRD5A1 and AR levels in the prostate glands of the testosterone-induced rat BPH model.

BPH is a hormone-related disease, where androgen signaling through its cognate receptor is known to play a pivotal role by promoting the proliferation of epithelial cells^10^. DHT, which is converted from testosterone by 5α reductases, is a more potent androgen than testosterone to stabilize and activate AR transcriptional activity, and consequently promote the pathogenesis of BPH in elderly men^17,38^. Consistently, the expression levels of AR in epithelial cells were significantly increased in BPH tissue compared with the normal prostate^16^.

Though currently α-blockers and 5αRIs are two major classes of drugs prescribed to treat BPH, which can relax the smooth muscles in the prostate/bladder neck and inhibit 5α-reductase to inhibit AR activity, respectively, these drugs demonstrate various types of side effects, including erectile dysfunction and cardiovascular risks^15–17^. Because herbal medicines possess similar efficacy but milder side effects compared with α-blockers and 5αRIs, recently more and more herbal extracts have been studied and used in clinical treatment for BPH as alternative medication.

Our study demonstrated that oral administration of Pao extract significantly reduced SRD5A1 and AR levels in prostate glands of the testosterone-induced BPH rat model. Given that Pao extract is an alkaloid-enriched extract, such *in vivo* effect may be due to the potential of these alkaloids. Previous studies have revealed that herbal extract of Leonuri herba alkaloids contains stachydrine and leonurine, which significantly reduced BPH symptoms, with the reduction of the levels of DHT and testosterone in the prostate homogenate, as well as the expression of FGF2 mRNA in the prostate^39^. This reinforced the notion that alkaloids may possess the inhibitory effects on SRD5A1 and AR. The Pao extract used in this study contains primarily flavopereirine which accounts for its most of its biological activity. Several other indole and β-carboline alkaloids are also present in minor amounts including alkaloid geissolosimine, geissospermine, geissoschizoline, and vellosiminol (also known as normacusine B), most of which have not yet well characterized^23,40,41^. We found that flavopereirine could inhibit the proliferation of RWPE-1 cells, and down-regulate the expression of SRD5A1 and AR. The anti-BPH efficacy of Pao extract, at least, was partially through flavopereirine.

In this study, we observed that Pao extract can significantly suppress testosterone -induced BPH, with the reduction of the thickness of the prostate epithelial cell layer and increase the lumen area. Consistently, we also found that another cell proliferation marker Cyclin D1 was also reduced in BPH/Pao group, compared to BPH group. Since activation of AR by binding with testosterone or DHT induce cell cycle gene expression, including Cyclin D1^42^, Pao extract may inhibit Cyclin D1 expression through reducing 5α reductase level and AR activity. Consistent with the suppressive effects of Pao extract on highly proliferative cells in BPH model, we and other group have demonstrated that Pao extract induced cell growth arrest and apoptosis in LNCaP and PC3 prostate cancer cells, as well as ovarian and pancreatic cancer cells^25,26^. In addition, Pao extract has been shown to inhibit multiple signaling pathways other than hormone-related signaling, such as Wnt/β-catenin and NFκB signaling in various cancer cells^27,29^. Notably, there is a causal link between prostatic inflammation and BPH development by epidemiological study^43,44^. Since Pao extract may possess anti-inflammation activity, probably through the inhibition of NFκB activation, it may be worth further dissecting whether Pao extract can affect other pathways involved in BPH pathogenesis. At current stage, we have not yet tested the anti-BPH effects of Pao on the developed BPH, which might be intriguing to examine in future.

In summary, our results demonstrated that 20 mg/kg Pao extract decreased the prostate weight, and the levels of 5α reductase level, and AR in testosterone-induced rat BPH models, with the minimal effect on the body weight and sperm counts. These data indicated that Pao extract could be a promising herbal medicine for BPH treatment. Further studies on its clinical trial and safety in human are required.

## Materials and Methods

### Sample procurements

Pao Pereira extract containing 54% β-carboline alkaloids analyzed by high-performance liquid chromatography were kindly provided by Natural Source International, Ltd. (New York, NY) and a single batch of the extract was used for the whole BPH study. Flavopereirine perchlorate was purchased from ChromaDex Inc. (Cat #: ASB00006066, Irvine, CA)

### Experimental animals and maintenance conditions

All the male *Sprague-Dawley* rats, weighing 180–200 g, were obtained from the Beijing Vital River Laboratory and Animal Technology Co., Ltd. (Beijing, China) and housed under regular conditions of temperature and a 12 h light/dark cycle with the supplement of standard laboratory diet and water ad libitum. The animal protocol was approved by the Administrative Panel on Laboratory Animal Care of Clinical College of Nanjing University (confirmation number: 2018GKJDWLS-03-002). All the experiments were carried out according to the international guidelines.

### BPH rat model

BPH rat model was produced as previously described^34,45–47^. In brief, 15 eight-week-old male SD rats weighing 180–200 g were anesthetized by intraperitoneal (*i.p*.) injection of sodium pentobarbital and castrated to exclude the influence of intrinsic testosterone. Control rats (n = 5) were sham operated. The castrated rats were randomly divided into three groups and generated to BPH by *i.p*. injections of 5 mg/kg testosterone propionate (Cat #: T101368, Aladdin Industrial Corporation, Shanghai, China) for 28 days. These groups were intragastric administrated with vehicle, Pao extract (20 mg/kg) or finasteride (10 mg/kg) for 28 days (Cat. #: HY-13635, MedChemExp, Shanghai, China), respectively. Finasteride was used as a positive control for the experimental drugs in the BPH studies. The rats were weighed weekly during the experiments. Under anesthesia by *i.p*. injections of deep sodium pentobarbital on day 29, prostates were removed, weighed, fixed in 4% formalin for histological and immunohistochemical (IHC) studies. The prostates were dissected and weighed to calculate the prostatic index (PI) using the following formula: PI = gross wet weight of prostate / weight of whole animal × 100%.

### Histological and immunohistochemical (IHC) staining

For histological examination, prostatic tissue specimens were fixed in 4% formalin, embedded in paraffin, sectioned at 5–6 μm and stained with hematoxylin and eosin (H&E). For IHC analysis, formalin-fixed, paraffin-embedded prostatic tissues were sectioned at 4–5 μm thickness. All sections were deparaffinized using 100% xylene, dehydrated with an ethanol gradient. Antigen retrieval was performed by autoclaving (100 °C for 5 min in 1 mM EDTA, pH 7.8). Incubation with primary antibodies against PCNA and androgen receptor (AR) were performed overnight at 4 °C. Information on the antibodies dilution were listed in Table 1. After washing with pH 7.4 phosphate-buffered saline (PBS), the sections were then incubated with a secondary antibody for 30 min at room temperature. Color development was performed with 3,3′-diaminobenzidine (DAB). Nuclei were lightly counterstained with hematoxylin. The positive cells were recognized by the appearance of brown staining. Five ventral prostate tissues of rat from each group were collected, and 5–6 fields/sample from maximum cross-sectional areas were analyzed. Expression levels were quantified in 5–6 fields/sample using Image J 1.47 v software (NIH, Bethesda, USA).

**Table 1:** Characteristics of the primary antibodies.

Primary antibodies	Host species	Supplier	IHC/WB	Dilution	Clone/code
AR	Rabbit	Santa Cruz	WB	1:200	N-20/sc-816
AR	Rabbit	Sigma	IHC	1:2,000	A9853
Cyclin B1	Mouse	Santa Cruz	WB	1:1,000	GNS1/sc-245
Cyclin D1	Rabbit	Santa Cruz	WB	1;1,000	sc-753
PCNA	Mouse	Santa Cruz	IHC	1:1,000	sc-56
PSA	Rabbit	Bioworld	WB	1:1,000	BS1302
SRD5A1	Rabbit	Proteintech	WB	1:1,000	26001-1-AP
Actin	Mouse	Sigma	WB	1:5,000	A1978

### Cell line

The nonmalignant epithelial cell line RWPE-1, which was purchased from the American Type Culture Collection (ATCC, Manassas, VA), was cultured in defined keratinocyte-SFM (10744019, Gibco, Grand Island, NY) at 37 °C under 5% CO_2_.

### Cell viability assay

The MTT assay was carried out as previous described^48^. Briefly, 24 h after 2,000 RWPE-1 cells were seeded in each well of 96-well plate, the cells were treated with Pao extract or flavopereirine with the indicated concentrations. At the end of the experiment, 10 μl of 5 mg/ml 3-(4,5-dimethylthiazol-2-yl) -2,5-diphenyltetrazolium bromide (MTT, Sigma-Aldrich, St. Louis, MO) was applied into each well for 4 h and the insoluble formozan in each well was dissolved in DMSO. The O.D. was measured at 490 nm wavelength with a reference wavelength of 680 nm by a microplate reader (BioTek Instruments, Winooski, VT, USA).

### Cell cycle analysis

RWPE-1 cells were seeded in the 60 mm plates and treated with Pao extract for 48 h or flavopereirine for 24 h. Cells were trypsinized and washed with ice-cold PBS, followed by the fixation in 75% ethanol for 1 h at 4 °C. The cells were washed with PBS once and incubated with 10 mg/ml RNase A (Sigma-Aldrich) and propidium iodide (PI, A601112, Sangon Biotech, Shanghai, China) in 1.0 ml PBS at 37 °C for 30 min to stain cells. The cell cycle analyses were performed using a FACScan Calibur flow cytometer (Becton Dickinson, San Jose, CA). Each cell cycle phase was analyzed by FlowJo software (FlowJo LLC, USA).

### Western blotting (WB)

Prostate tissues were homogenized by using the tissue homogenizer with cold RIPA buffer, followed by the centrifugation at 12,000 rpm for 30 min at 4 °C to remove tissue debris. RWPE-1 cells were harvested after the treatment with Pao extract or flavopereirine at the indicated concentration. Bradford method was used to measure protein concentration. After protein samples were separated by sodium dodecyl sulfate-polyacrylamide gel electrophoresis (SDS-PAGE), proteins were transferred from the gel to the PVDF membrane, followed by blocking in PBST buffer supplemented with 5% non-fatted milk for 1 h at room temperature. The membranes were incubated with primary antibodies against AR, Cyclin B1, Cyclin D1, PSA or SRD5A1 at 4 °C overnight, which were shown in Table 1. After washed with PBST three times, the membranes were incubated with secondary antibodies. The membrane was exposed to a Tanon Luminescent Imaging Workstation after using Tanon High-sig ECL Western Blotting Substrate (Cat #: 180-5001, Tanon Science & Technology Co., Ltd, Shanghai, China). Actin was used as a control of an equal loading. The chemiluminescence intensity of protein signals were quantified by the Image J software (NIH).

### Epididymal sperm count analysis

15 eight-week-old male SD rats were randomly divided into three groups. The three group rats were intragastric administrated with vehicle, Pao extract (20 mg/kg) or finasteride (10 mg/kg) once daily for 4 weeks, respectively. After that, these rats were sacrificed on day 29. The cauda epididymis was removed, minced and placed into 4 ml of normal saline for 20 min at 37 °C to obtain sperm suspension. Then the sperm suspension was diluted to 100 times with normal saline and gently mixed. Following that, the sperm count was calculated under an optical microscope.

### Statistical analysis

Semi-quantification was performed by Image J software. Normal distribution was confirmed or assumed (for n < 5) and further analyzed with unpaired *t*-test to determine the differences between each group and p < 0.05 was considered as statistically significant differences. All data were expressed as the mean ± SD. All statistical analyses were performed by using GraphPad Prism software (GraphPad Software).

## Supplementary information

Supplementary Information

## Data Availability

All data generated or analyzed during this study are included in this published article.
