# Supplementary material for: Pao Pereira Extract Attenuates Testosterone-Induced Benign Prostatic Hyperplasia in Rats by inhibiting 5α-Reductase
Source: Sci Rep. 2019 Dec 23;9:19703. doi: 10.1038/s41598-019-56145-z (PMC6928012; doi:10.1038/s41598-019-56145-z)
Supplement: Supplementary file 1 — Supplementary Information [file 41598_2019_56145_MOESM1_ESM.docx]

Pao Pereira Extract Attenuates Testosterone-Induced Benign Prostatic Hyperplasia in Rats by inhibiting 5α-Reductase

**Jiakuan Liu^1,†^, Tian Fang^2,†^, Meiqian Li^1,†^, Yuting Song^1^, Junzun Li^1^, Zesheng Xue^1^, Jiaxuan Li^1^, Dandan Bu^1^, Wei Liu^1^, Qinghe Zeng^3,4^, Yidan Zhang^1,5^, Shifeng Yun^2,^*, Ruimin Huang^3,4,^*, Jun Yan^1,^***

^1^ State Key Laboratory of Pharmaceutical Biotechnology and MOE Key Laboratory of Model Animals for Disease Study, Model Animal Research Center of Nanjing University, Nanjing 210061, Jiangsu, China;

^2^ Department of Comparative Medicine, Jinling Hospital, Nanjing University School of Medicine, Nanjing 210002, Jiangsu, China;

^3^ Shanghai Institute of Materia Medica, Chinese Academy of Sciences, Shanghai 201203, China;

^4^ University of Chinese Academy of Sciences, Beijing 100049, China;

^5^ Department of Bioscience and Bioengineering, School of Chemistry and Life Science, Jinling College of Nanjing University, Nanjing 210061, Jiangsu, China.

***** Correspondence: rmhuang@simm.ac.cn; Tel.: +86-021-5080-5853 (R.H.); yunshifeng1@163.com, Tel.: +86-025-8086-0320 (S.Y.); yanjun@nju.edu.cn; Tel.: +86-025-5864-1535 (J.Y.)

† These authors contributed equally to this work.


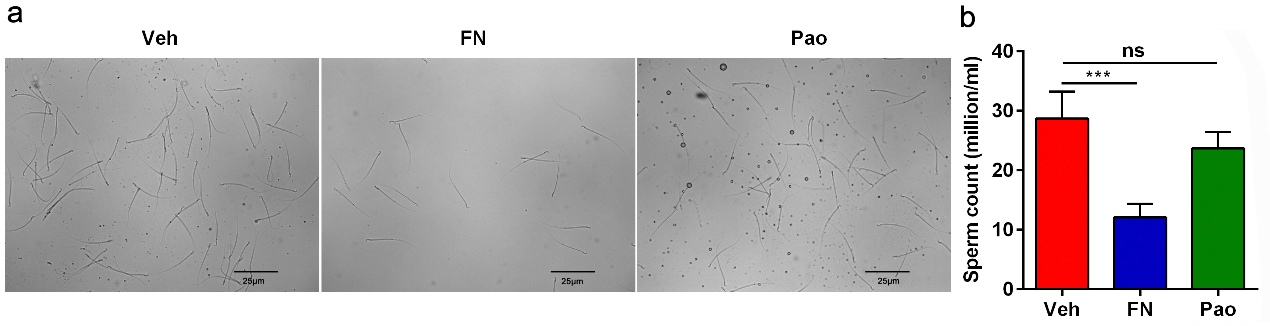


**Supplementary Figure S1. The effect of Pao extract and finasteride on epididymal sperm numbers of rats.** (**a**) The image of sperm number treated with Vehicle (Veh), finasteride (FN) and PAO extract (Pao). (**b**) Quantification of epididymal sperm number. The values were presented as the mean ± SD. n = 5. ns, not significance; ***, p < 0.001.





**Supplementary Figure 2. Uncropped images of Fig. 3c, 4c, 5e, 6a, 7a and 8a.**
